# Applicability of TB-LAMP test for diagnosis of pulmonary TB among HIV-positive individuals

**DOI:** 10.5588/ijtldopen.24.0410

**Published:** 2025-01-01

**Authors:** B. Muriithi, M.M. Bundi, T. Moriyasu, A. Ahmed, M. Mwamzuka, M. Karama, S. Kaneko

**Affiliations:** ^1^ Nairobi Research Station, Nagasaki University Institute of Tropical Medicine (NUITM)-Kenya Medical Research Institute (KEMRI) Project, Nairobi, Kenya;; ^2^ Kenya Medical Research Institute, KEMRI Graduate School, Nairobi, Kenya;; ^3^ Office for International Relations, Nagasaki University, Nagasaki, Japan;; ^4^ Bomu Hospital, Mombasa, Kenya;; ^5^ African Medical and Research Foundation (AMREF) Health Africa Ethics and Scientific Research Committee, AMREF Health Africa, Nairobi, Kenya;; ^6^ Department of Ecoepidemiology, Institute of Tropical Medicine, Nagasaki University, Nagasaki, Japan.

**Keywords:** diagnosis, HIV, tuberculosis, pulmonary TB, loop-mediated isothermal amplification

## Abstract

**BACKGROUND:**

The loop-mediated isothermal amplification for TB (TB-LAMP) assay is more cost-effective and accessible than the Xpert^®^ MTB/RIF assay. This study aimed to evaluate the diagnostic performance of the TB-LAMP assay in individuals with and without HIV infection.

**METHODS:**

Patients aged ≥15 years presenting with symptoms of TB were included in the study. The TB-LAMP assay was performed alongside routine TB diagnostic methods, including the Xpert assay and smear microscopy, to evaluate discrepancies in test results and associated factors.

**RESULTS:**

A total of 903 patients were enrolled in the study. The positive percentage agreement for smear microscopy and TB-LAMP compared with the Xpert assay was respectively 54.3% (95% CI 46.6–61.8) and 76.6% (95% CI 69.9–82.6). Among HIV-positive individuals, the positive percentage agreement was 42.5% (95% CI 32.9–52.4) for smear microscopy and 68.9% (95% CI 59.1–77.5) for TB-LAMP. Factors such as age >60 years (adjusted OR 3.29, 95% CI 0.32–33.83), loss of appetite (aOR 0.30, 95% CI 0.13–0.70), and HIV-positive status (aOR 3.29, 95% CI 1.12–9.63) were associated with discrepancies between TB-LAMP and Xpert results.

**CONCLUSIONS:**

TB-LAMP demonstrated better agreement with the Xpert assay compared with smear microscopy in detecting TB among HIV-positive patients, suggesting that TB-LAMP could effectively replace smear microscopy.

## RÉSUMÉ

**CONTEXTE:**

Le test d'amplification isotherme en boucle pour la TB (TB-LAMP, pour l’anglais « *loop-mediated isothermal amplification for TB* ») présente des avantages en termes de coût et d'accessibilité par rapport au test Xpert^®^ MTB/RIF. Cette recherche avait pour objectif d'analyser l'efficacité diagnostique du test TB-LAMP chez des individus, qu'ils soient infectés ou non par le VIH.

**MÉTHODES:**

Les patients âgés de ≥15 ans présentant des symptômes de TB ont été intégrés à l'étude. Le test TB-LAMP a été effectué en même temps que les méthodes de diagnostic habituelles de la TB, telles que le test Xpert et l'analyse microscopique des échantillons, dans le but d'examiner les différences dans les résultats des tests ainsi que les facteurs qui y sont liés.

**RÉSULTATS:**

Un total de 903 patients a été inclus dans l'étude. Le taux de concordance positive pour la microscopie à frottis et la TB-LAMP par rapport au test Xpert était respectivement de 54,3% (IC à 95% 46,6–61,8) et de 76,6% (IC à 95% 69,9–82,6). Parmi les individus séropositifs, le taux de concordance positive s'élevait à 42,5% (IC à 95% 32,9–52,4) pour la microscopie à frottis et à 68,9% (IC à 95% 59,1–77,5) pour la TB-LAMP. Des éléments tels que l'âge supérieur à 60 ans (OR ajusté [ORa] 3,29 ; IC à 95% 0,32–33,83), la perte d'appétit (ORa 0,30 ; IC à 95% 0,13–0,70) et la séropositivité (ORa 3,29 ; IC à 95% 1,12–9,63) ont été identifiés comme des facteurs liés aux divergences entre les résultats de la TB-LAMP et ceux du Xpert.

**CONCLUSIONS:**

La méthode TB-LAMP a démontré une concordance supérieure avec le test Xpert en comparaison avec la microscopie de frottis pour la détection de la TB chez les patients séropositifs, ce qui indique que la TB-LAMP pourrait constituer une alternative efficace à la microscopie de frottis.

TB is a major global public health concern. Persons living with the HIV are at risk of developing TB, the most prevalent opportunistic infection. Despite the availability of prevention and treatment options, morbidity rates remain high in countries with a high disease burden.^1,2^ A significant barrier to reducing TB is the continuous transmission from untreated individuals with TB due to suboptimal diagnosis, with or without HIV. Therefore, accurate and timely diagnosis of TB is critical, and the application of a comprehensive diagnostic tool is a priority.^3^

Sputum smear microscopy and culture techniques are effective diagnostic approaches that have been used for many years. However, the sensitivity of microscopy is low, particularly in paucibacillary disease characteristic of HIV-TB co-infection, and culture takes a long time to yield results.^4,5^ Despite being diagnostic standards, these methods have not been able to meet the needs for early and rapid diagnosis. Therefore, an ideal TB diagnostic test would be a test with high sensitivity and specificity and low turnaround time, yet inexpensive and cost-effective.

In recent decades, nucleic acid amplification diagnostic tests and immunological assays have been introduced to address the limitations of direct culture and sputum smear microscopy.^6–11^ However, the financial burden associated with the introduction and maintenance of these technologies can be substantial.^12^ While immunological assays are generally more cost-effective, their sensitivity and specificity are not sufficient for them to be used as first-line diagnostic tools.^13^ It is also crucial to note that both methodologies demonstrate inconsistent sensitivity and specificity, particularly in paediatric populations and HIV-infected patients.^13,14^

Xpert^®^ MTB/RIF (Xpert; Cepheid, Sunnyvale, CA, USA) is the most widely used modern TB diagnostic assay. The WHO has approved it as an initial diagnostic test, replacing microscopy. It is used worldwide, including in countries with high rates of TB.^15^ However, it requires significant financial resources because of the high initial cost of the instrument and the high maintenance cost of cartridges. Furthermore, its dependence on specialised infrastructure constrains its applicability in more decentralised care settings, which may ultimately undermine its fundamental objective of enhancing patient access to prompt and precise diagnosis.

Loop-mediated isothermal amplification for TB (TB-LAMP) is an alternative nucleic acid amplification test.^16^ This technology integrates DNA extraction and purification with LAMP amplification at isothermal temperatures, making it highly sensitive and rapid.^16,17^ TB-LAMP is 7.1–13.2% more sensitive than microscopy and 90–100% more specific than microscopy.^18^ A systematic review found that it is as accurate as Xpert, and a follow-up study in children showed that TB-LAMP is as sensitive as other molecular assays.^19,20^ It is simple, affordable, has a shorter turnaround time and does not require specialised equipment. It can be used in peripheral clinics and regions with limited resources. Its use is expected to increase in areas with a high prevalence of HIV, but the performance of the LAMP assay in HIV-positive patients is unclear.^21^

This study aims to evaluate the accuracy of TB-LAMP for diagnosing TB in HIV-positive individuals, comparing TB-LAMP’s diagnostic performance against sputum smear microscopy and Xpert.

## METHODS

### Study site and study participants

This study was conducted at Bomu Hospital in Mombasa County, Kenya, from August 2021 to December 2022, Mombasa County being among counties with high rates of TB and HIV.^22,23^ Bomu Hospital is located in Changamwe sub-county, with outpatient clinics in the Likoni and Kisauni sub-counties. The hospital has comprehensive care centres and an accredited TB testing centre. Patients aged ≥15 years with at least one of the typical TB symptoms were recruited consecutively until the study ended. Recruitment was conducted in the outpatient departments or comprehensive care clinics. Patients who agreed to participate signed an informed consent form and were enrolled.

### Sputum sample collection

The study utilised part of a sputum sample provided by the patient for routine diagnosis of TB using Xpert. Laboratory technologists first explained to the patient how to produce a good quality sputum sample, after which they were requested to produce at least 2 ml sputum samples.

### TB-LAMP assay

The TB-LAMP assay was performed according to the manufacturer’s recommendations (Eiken Chemical Company, Tokyo, Japan). A Loopamp PURE DNA extraction kit was used for extraction of *Mycobacterium tuberculosis* DNA. A total of 60 µL of the sample were collected from the most purulent spot in the specimen and placed in a heating tube. The sputum sample was mixed with the DNA extraction solution by inverting the tube three times. The heating tube was incubated at 90°C in an LF-160 incubator to facilitate the lysis of bacterial cells and the subsequent release of DNA into the solution. Subsequently, the heating tube was adjoined onto an adsorbent tube containing adsorbent powder for DNA purification. Thirty microlitres of purified DNA were collected in a reaction tube containing LAMP reagents. After mixing the reagents with the DNA solution, the mixture was incubated at 67°C for 40 min for DNA amplification using LAMP. The reaction products were visualised under ultraviolet light and the results were recorded.

### Xpert MTB/RIF assay

The Xpert assay was performed according to the manufacturer’s instructions as part of routine diagnostic performance in the hospital. The surface of the falcon tube containing the sample was decontaminated in a biosafety cabinet. The sputum was then treated with sodium hydroxide and a sample reagent was added to the sample at a 2:1 ratio. After 15 min of incubation, the treated sample was transferred into a cartridge and then loaded onto GeneXpert instruments to complete the automated steps. Xpert cartridges were used from August 2021 to October 2021 and Xpert^®^ MTB/RIF Ultra (Ultra; Cepheid) cartridges from February 2022 to December 2022.

### Sputum smear microscopy

A smear of uniform thickness was made on a glass slide and examined for *M. tuberculosis* using Ziehl-Neelsen’s stain.^24^

### Statistical analysis

Categorical variables were summarised using descriptive statistics and presented as percentages. McNemar’s test was used to test for differences in positivity by Xpert and TB-LAMP to assess the performance of TB-LAMP among persons living with HIV. Positive and negative concordance or discordance status of TB-LAMP and sputum smear with Xpert results were summarised, and the concordance percentages were calculated with the 95% confidence intervals (CIs), applying the exact binomial confidence intervals method. The test of proportions was used to establish the statistical significance of the differences in concordance percentages. In addition, analyses of determinants for discordance for TB-LAMP-negative patients against Xpert-positives were conducted using age, sex, cough, duration of cough, chest pain, loss of appetite, nocturnal sweating, weight loss, fever, and HIV status as explanatory variables by logistic regression and calculated crude odds ratios (ORs) and adjusted ORs (aORs). In calculating aORs, variables were selected using a backward stepwise method with a significance level (*P*-value) of 0.05 for a variable to be removed from the model. Analyses were conducted using STATA v15 software (StataCorp, College Station, TX, USA).

### Ethical considerations

Ethical approval for this study was obtained from the African Medical and Research Foundation Ethics and Science Review Committee, Nairobi, Kenya (AMREF-ESRC: P848-2020) and the Ethical Committee of the Institute of Tropical Medicine, Nagasaki University, Nagasaki, Japan (Approval No.: 200619238-2). Written informed consent was obtained from all patients who agreed to participate in the study.

## RESULTS

A total of 903 patients participated in this study. More than half (62.1%) of the patients were female. Most patients (95.6%) presented with a cough lasting approximately 2 weeks (Table 1). In total, there were 173 HIV-negative, 646 HIV-positive, and 84 individuals with unknown HIV status. The positivity of TB by Xpert was 19.4% and 14.9% according to TB-LAMP among all patients. The prevalence of HIV among potential TB patients when all presumptive TB patients were enrolled, regardless of their HIV status, was 71.5%. TB positivity on Xpert was higher than by TB-LAMP among persons living with HIV (Table 1). Detection rate differences and McNemar’s test results by Xpert and TB-LAMP among HIV-negative and HIV-positive persons are shown in Table 2. The difference in detection rate was statistically significant among HIV-positive individuals.

**Table 1.** Demographic and clinical characteristics of patients according to HIV status.

Characteristics	HIV-negative *n* (%)	HIV-positive *n* (%)	Unknown HIV status *n* (%)	Total *n* (%)
Sex				
Male	93 (53.8)	202 (31.3)	47 (56.0)	342 (37.9)
Female	80 (46.2)	444 (68.7)	37 (44.0)	561 (62.1)
Age category, years				
<20	13 (7.6)	34 (5.3)	12 (14.8)	59 (6.6)
21–35	58 (33.7)	144 (22.4)	22 (27.2)	224 (24.97)
36–60	82 (47.7)	417 (64.7)	38 (46.9)	537 (59.9)
>61	19 (11.1)	49 (7.7)	6 (11.1)	77 (8.6)
Cough				
No cough	7 (4.0)	29 (4.5)	0 (0.0)	36 (4.0)
Cough	166 (96.0)	617 (95.5)	80 (95.2)	863 (95.6)
Missing	0 (0.0)	0 (0.0)	4 (0.0)	4 (4.0)
Duration of cough, weeks				
<1	8 (4.8)	74 (12.0)	9 (11.3)	91 (10.6)
1	38 (22.9)	128 (20.8)	18 (22.5)	184 (21.4)
2	40 (24.1)	200 (32.5)	23 (28.7)	263 (30.5)
3	17 (10.4)	62 (10.1)	8 (10.0)	87 (10.1)
>3	63 (37.9)	151 (24.5)	22 (27.5)	236 (27.4)
Chest pain				
No	47 (27.2)	210 (32.5)	22 (26.2)	279 (30.9)
Present	126 (72.8)	436 (67.5)	62 (73.8)	624 (69.1)
Loss of appetite				
No	97 (56.1)	409 (63.3)	47 (55.9)	553 (61.2)
Present	76 (43.9)	237 (36.7)	37 (44.1)	350 (38.8)
Night sweat				
No	107 (61.8)	448 (69.4)	49 (58.3)	604 (66.9)
Present	66 (38.2)	198 (30.6)	35 (41.7)	299 (33.1)
Weight loss				
No	105 (60.7)	376 (58.2)	56 (66.7)	537 (59.5)
Present	68 (39.3)	270 (41.8)	28 (33.3)	366 (40.5)
Fever				
No	84 (48.5)	398 (61.6)	49 (58.3)	531 (58.8)
Present	89 (51.5)	248 (38.4)	35 (41.7)	372 (41.2)
Xpert MTB/RIF result				
Negative	125 (72.3)	540 (83.6)	63 (75.0)	728 (80.6)
Positive	48 (27.7)	106 (16.4)	21 (25.0)	175 (19.4)
TB-LAMP result				
Negative	130 (75.1)	573 (88.7)	65 (77.4)	768 (85.1)
Positive	43 (24.9)	73 (11.3)	19 (22.6)	135 (14.9)
Smear test				
Negative	139 (80.3)	600 (92.9)	68 (80.9)	807 (89.4)
Positive	34 (19.6)	45 (7.0)	16 (19.1)	95 (10.5)
Missing	0 (0.0)	1 (0.2)	0 (0.0)	1 (0.1)
Recruitment inclusion*				
Only HIV cases	34 (19.7)	403 (62.4)	9 (10.7)	446 (49.4)
All suspected	139 (80.3)	243 (36.7)	75 (89.3)	457 (50.6)

* Due to insufficient Xpert MTB/RIF cartridges, there were periods when only HIV-positive participants were registered for the study.

TB-LAMP = loop-mediated isothermal amplification for TB.

**Table 2.** Detection rate by Xpert MTB/RIF and TB-LAMP among HIV-negative and HIV-positive persons.

	TB-LAMP	Xpert MTB/RIF	*P*-value
	Detection rate *n* (%)	Detection rate *n* (%)	
HIV-negative	43 (24.9)	48 (27.7)	0.059
HIV-positive	73 (11.3)	106 (16.4)	<0.001

*McNamar’s test used for statistical analysis to show the differences in positivity by Xpert MTB/RIB and TB-LAMP.

TB-LAMP = loop-mediated isothermal amplification for TB.

Positive and negative percentage agreement between Xpert and smear test/TB-LAMP are shown in Table 3. Positive percentage agreement for smear test and TB-LAMP compared with Xpert assay was 54.3% (95% CI 46.6–61.8) and 76.6% (95% CI 69.9–82.6) for all patients. Among persons living with HIV, positive percentage agreement was respectively 42.5% (32.9–52.4) and 68.9% (95% CI 59.1–77.5). Positive percentage agreement of smear test and TB-LAMP with Xpert were statistically different among all populations (Table 3). Negative agreement percentages ranged between 99.2% and 100% for both diagnostic tests compared with Xpert assay results.

**Table 3.** Agreement between Xpert MTB/RIF and smear test and TB-LAMP among all patients and persons living with and without HIV.

		Smear test		TB-LAMP results		
	Xpert cases *n*	Agreement *n* (%)	95% CI	Agreement *n* (%)	95% CI	*P*-value
All patients						
Positive	175	95 (54.3)	46.6–61.8	134 (76.6)	69.6–82.6	<0.001
Negative	728*	727 (100.0)	99.5–100.0	727 (99.9)	99.2–100.0	0.394
Persons without HIV^†^						
Positive	48	34 (70.8)	55.9–83.0	42 (87.5)	74.8–95.3	0.044
Negative	125	125 (100.0)	97.1–100.0	124 (99.2)	95.6–100.0	0.317
Persons living with HIV^†^						
Positive	106	45 (42.5)	32.9–52.4	73 (68.9)	59.1–77.5	0.0001
Negative	540*	539 (100.0)	99.3–100.0	540 (100.0)	99.3–100.0	

* One without a smear test. For agreement percentage calculations, 727 Xpert-negative cases were used.

† Among 903 patients, 84 with unknown HIV status were removed from the tables.

TB-LAMP = loop-mediated isothermal amplification for TB.

Factors associated with discrepancies between Xpert and TB-LAMP assays among Xpert positive cases (*n* = 175) by logistic regression analysis are shown in Table 4. Univariate analysis revealed fewer discordant results in patients with loss of appetite (OR 0.25, 95% CI 0.12–0.54), weight loss (OR 0.42, 95% CI 0.21–0.86), and fever (OR 0.45, 95% CI 0.22–0.93). Conversely, a higher discrepancy was noted among HIV-positive patients, with an OR of 3.2 (95% CI 1.22–8.17) compared to their HIV-negative counterparts.

**Table 4.** Result of logistic regression analysis for factors related to disagreement of the results between Xpert MTB/RIF and TB-LAMP among Xpert-positive cases (*n* = 175).

Characteristics	OR (95% CI)	*P*-value	aOR (95% CI)	*P*-value
Sex				
Male	1			
Female	1.56 (0.77–3.16)	0.212		
Age category, years				
<20	1		1	
21–35	0.23 (0.04–1.24)	0.087	0.31 (0.05–1.88)	0.203
36–60	0.39 (0.08–1.86)	0.081	0.43 (0.08–2.35)	0.331
>61	1.67 (0.23–12.22)	0.615	3.29 (0.32–33.83)	0.317
Cough				
No cough	1			
Cough	0.28 (0.04–2.07)	0.214		
Missing	2.0 (0.09–44.35)	0.661		
Duration of cough, weeks				
<1	1			
1	0.33 (0.06–1.76)	0.196		
2	0.38 (0.08–1.79)	0.22		
3	0.16 (0.02–1.00)	0.05		
>3	0.21 (0.05–0.96)	0.045		
Chest pain				
No	1			
Present	0.76 (0.36–1.63)	0.484		
Loss of appetite				
No	1		1	
Present	0.25 (0.12–0.54)	<0.001	0.30 (0.13–0.70)	0.005
Night sweat				
No	1			
Present	0.74 (0.37–1.50)	0.408		
Weight loss				
No	1			
Present	0.42 (0.21–0.86)	0.018		
Fever				
No	1			
Present	0.45 (0.22–0.93)	0.032		
HIV status				
Negative	1		1	
Positive	3.16 (1.22–8.17)	0.017	3.29 (1.12–9.63)	0.03
Unknown	0.73 (0.14–3.00)	0.723		

TB-LAMP = loop-mediated isothermal amplification for TB; OR = odds ratio; CI = confidence interval; aOR = adjusted OR.

In the multivariate analysis, following a stepwise variable selection process, age, loss of appetite, and HIV status remained in the model. Reduced discordance between TB-LAMP and Xpert assays was observed in patients with a loss of appetite (aOR 0.30, 95% CI 0.13–0.70), and higher discordance among HIV-positive patients (aOR 3.29, 95% CI 1.06–9.34) compared to HIV-negative individuals.

The proportions of discrepancies of the results between Xpert and TB-LAMP/smear test are shown in the Figure by HIV status and Xpert diagnostic category. The discrepancy proportions were nearly identical at higher quantification volumes of *M. tuberculosis* on Xpert (high and medium); however, the discrepancy rates widened as the bacilli volume potentially decreased among persons living with HIV. This trend was more significant for the smear tests than for the TB-LAMP results.

**Figure.**
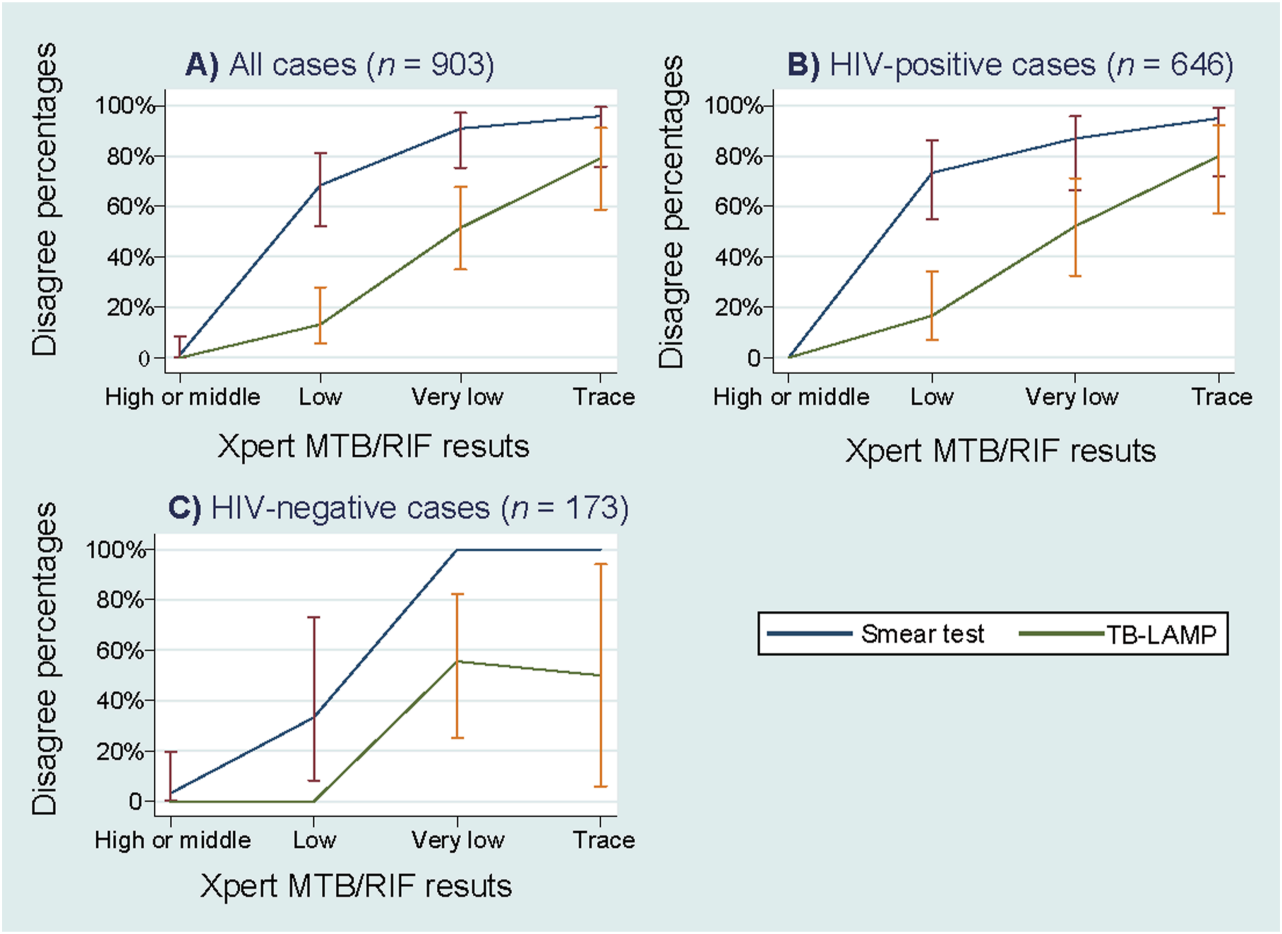
Discrepancy percentages between the Xpert MTB/RIF and TB-LAMP/smear test results according to the diagnostic intensity categories of Xpert MTB/RIF and HIV status. TB-LAMP = loop-mediated isothermal amplification for TB.

## DISCUSSION

In this study, TB-LAMP and sputum smear microscopy were evaluated in comparison with the Xpert assay as a reference standard, given the advantages of TB-LAMP over Xpert and smear microscopy.^25^ This study showed a significant discrepancy between TB-LAMP and Xpert results in patients living with HIV, especially those with low sputum bacterial counts according to Xpert. This may be due to the fact that TB infection in HIV-infected patients presents with low bacillary count and may be difficult to detect using the TB-LAMP method.^26–28^ When compared to smear microscopy, the positive agreement rate with Xpert was higher, suggesting that TB-LAMP is more accurate than smear microscopy. The higher concordance of TB-LAMP compared to smear testing in both HIV-positive and HIV-negative individuals implies that TB-LAMP can be used as an alternative to smear microscopy if a more accurate diagnosis is sought.

On the other hand, the possibility of false-negatives on TB-LAMP should be considered, given the discrepancies in results with Xpert. In particular, since the discrepancies are more common in patients with less pronounced TB symptoms, consideration should be given to the diagnosis of patients with fewer symptoms, especially HIV-infected patients. If the Xpert results are correct, repeated TB-LAMP testing or switching to Xpert should be considered for such cases, although TB-LAMP has proven to be quite accurate relative to Xpert and sputum culture, highlighting its potential as an alternative or adjunctive test.^19,29,30^ In any case, it is feasible to improve the accuracy of TB diagnosis in end-care facilities by replacing smear microscopy with TB-LAMP.

Discrepancy between TB-LAMP and Xpert may be explained by technical differences between the two tests. The Xpert test is designed to process sputum specimens of 1 ml or more, while the TB-LAMP method uses only 60 µL of sputum specimen. Sensitivity of TB-LAMP may be reduced by the low specimen volume. Furthermore, the TB-LAMP method is susceptible to inter-operator variability due to sampling bias and may under-detect bacilli, especially in low bacillary disease or poor-quality sputum specimens.^31^ Moreover, the Xpert method has an additional liquefaction step to disperse bacillus clusters in the specimen, which improves specimen uniformity and sampling accuracy. In fact, Cheng et al. reported that the sensitivity and specificity of TB-LAMP were comparable to the Xpert assay when vortexing was used in the sample preparation step.^32^ Therefore, the discrepancy between Xpert and TB-LAMP results observed in this study may not be due to the sensitivity of TB-LAMP itself or the sensitivity of the TB-LAMP assay, but could be attributed to sample collection and volume.

The time between specimen collection and testing may have also contributed to discrepancy. Some specimens were collected at the satellite clinic and stored for less than 24 hours before being transported to the main clinic where the measurements were performed. This short storage time may have degraded the specimens, decreasing the amount of bacilli and possibly affecting detection by TB-LAMP.

The limit of detection (LOD) should also be considered: the LOD of Xpert is 131 colony-forming units (CFU)/ml, the LOD of Ultra is 15.6 CFU/mL, and the LOD of TB-LAMP is approximately 100 CFU/mL.^33–35^ Regarding the difference in sensitivity, Ultra has been reported to yield false-positive cases due to its high sensitivity.^36^ Therefore, there is a possibility of false positives in cases where Xpert was found to be positive at trace amounts and further verification is needed. Generally, prior studies have shown that TB-LAMP performs sub-optimally compared to the WHO standard.^37,38^ However, with improved methodology and procedures, it could be comparable to the Xpert assay. Further studies are needed to confirm this, including specimen manipulation procedures.

A limitation of this study is the use of Xpert as a reference standard. To obtain a more accurate assessment, instruments such as quantitative polymerase chain reaction (qPCR)with TB-LAMP or Xpert should be used. Nevertheless, comparison with Xpert, which is currently used as the standard TB diagnosis, is important for policy making.

In conclusion, the sensitivity of TB-LAMP was found to be reduced in comparison to Xpert in HIV-positive patients with low sputum bacterial counts. However, it is more accurate than smear microscopy and less influenced by the examiner’s experience. Additionally, false-negative results among HIV-positive individuals without symptoms should be considered when using TB-LAMP.
